# Low-temperature stress modulates pollen tube growth through temperature-dependent multi-level regulatory mechanisms in *Camellia sinensis*

**DOI:** 10.1007/s00497-026-00539-3

**Published:** 2026-05-08

**Authors:** Sena Acar, Aslıhan Çetinbaş-Genç

**Affiliations:** 1 Institute of Pure and Applied Sciences, Marmara University, 34722 Istanbul, Türkiye; 2 Department of Biology, Faculty of Science, Marmara University, 34722 Istanbul, Türkiye

**Keywords:** Antioxidant capacity, Cell wall, Heat shock protein, Low temperature, Pollen tube, Sucrose synthase

## Abstract

Low temperature is a major environmental stress factor that limits male reproductive success in plants; however, the mechanistic basis of pollen responses to different degrees of low temperature stress remains poorly understood. In this study, we investigated the cytological, biochemical, and molecular responses of tea (*Camellia sinensis*) pollen grains to low-temperature stress by germinating pollen in vitro at 15, 10, and 5 °C. Although pollen germination rate and pollen tube length were reduced under all low-temperature treatments compared with the control, the underlying regulatory responses differed significantly with the severity of low-temperature stress. At 15 °C, decreases in non-enzymatic antioxidants and stress-related proteins indicated an overall metabolic weakening, while the concomitant accumulation of callose, cellulose, and methyl-esterified pectins reflected an early structural adjustment of the pollen tube cell wall. At 10 °C, the increasing stress load was partially counterbalanced by the induction of enzymatic antioxidant activities, and continued cell wall reinforcement identified this temperature as a transitional state between metabolic limitation and coordinated defense activation. In contrast, exposure to 5 °C resulted in pronounced metabolic suppression, together with a shift of stress-related proteins toward membrane fractions and enhanced deposition of callose, cellulose, and particularly de-esterified acidic pectins, leading to increased cell wall rigidity and mechanical restriction of pollen tube elongation. Overall, this study demonstrates that low-temperature stress constrains pollen tube growth not through a single limiting factor but via a temperature-dependent reorganization of interconnected cytological, biochemical, and molecular mechanisms.

**Supplementary Information:**

The online version contains supplementary material available at 10.1007/s00497-026-00539-3.

## Key Message

Low temperature reshapes tea pollen tube growth by coordinating cell wall remodeling, antioxidant defenses, and stress protein dynamics, revealing temperature specific mechanisms underlying reproductive stress tolerance.

**Supplementary Information:**

The online version contains supplementary material available at 10.1007/s00497-026-00539-3.

## Introduction

Tea (*Camellia* spp.) is an evergreen, perennial plant belonging to the family Theaceae (Pascoa et al. 2019). Although tea is predominantly cultivated in tropical and subtropical regions, it is frequently exposed to low temperature stress during winter and early spring, which restricts both vegetative and generative development and leads to significant economic losses in the tea industry (Wang et al. 2022). In recent years, the northward expansion of tea cultivation areas, together with abrupt temperature fluctuations associated with climate change, has further increased the susceptibility of tea plants to cold stress (Zhou et al. 2025).

Prolonged exposure to low temperature is known to cause damage to DNA, proteins, lipids, and membrane structures in plants, thereby adversely affecting morphological, physiological, and biochemical processes (Heidarvand et al. 2010; Ritonga and Chen 2020). However, the majority of existing studies have primarily focused on vegetative growth, while the effects of low temperature stress on generative development have been addressed only to a limited extent (Guerra et al. 2015; Petruccelli et al. 2022). In contrast, generative development is considerably more sensitive to environmental stresses, and disruptions occurring during these processes directly affect crop yield and quality (Llorens et al. 2015). Although leaf production is of primary economic importance in tea cultivation, the expanding applications of tea oil derived from seeds and fruits have further increased the significance of generative processes in tea plants (Sahari and Amooi 2013). Therefore, understanding the sensitivity of generative processes that determine fruit set to environmental stresses is of critical importance. One of the most important stages of generative development in tea plants is pollination. However, exposure to low temperature stress during this process can inhibit pollen germination and pollen tube elongation, thereby negatively affecting fertilization and fruit set. The high sensitivity of pollen grains, the fundamental component of pollination, to environmental stresses has made them reliable biological indicators for assessing various stress factors in recent years (Malayeri et al. 2011; Ramirez et al. 2022). In this context, elucidating the cytological, biochemical and molecular responses developed by pollen under fluctuating climatic conditions is of critical importance for plant reproductive biology (Aloisi et al. 2022).

Under environmental stress conditions, pollen performance is commonly evaluated through parameters such as viability, germination rate, and pollen tube length (Liu et al. 2024). Although a strong relationship exists between pollen viability and germination rate, pollen tube elongation is regulated by more complex processes involving multiple cellular mechanisms (Scholz et al. 2020). Pollen tube elongation depends on the delicate balance between turgor pressure and the mechanical properties of the tube cell wall (Dumais 2021). This balance is maintained by a more flexible cell wall structure predominantly composed of methyl-esterified pectins at the apical region, and a more rigid cell wall organization in the subapical neck region, enriched in de-esterified acidicpectins as well as callose and cellulose. Accordingly, this organization is associated with the non-uniform distribution of pectin, callose, and cellulose components along the pollen tube wall (Hepler et al. 2013; Çetinbaş-Genç et al. 2022). Environmental stresses can adversely affect pollen tube elongation by altering the spatial distribution of these components along the tube; therefore, changes in pollen tube wall components are widely used as important indicators for evaluating stress effects (Parrotta et al. 2016; Fang et al. 2016).

Once pollen metabolism is activated during germination, pollen tubes become highly sensitive to environmental conditions (Goel et al. 2023). Despite the importance of pollen performance for plant reproduction, the mechanisms underlying pollen responses to low temperature stress remain poorly understood. Stress conditions cause damage to biomolecules in pollen grains and pollen tubes, leading to the excessive accumulation of reactive oxygen species (ROS) (Xie et al. 2022). ROS, which are of critical importance for pollen tube elongation, represent the balance between oxidative stress and tube growth and indirectly regulate elongation through the cytosolic Ca²⁺ gradient and actin filament organization (Scholz et al. 2020). Therefore, the maintenance of ROS homeostasis in pollen tubes is closely associated with enzymatic antioxidant systems, such as superoxide dismutase (SOD), catalase (CAT), and ascorbate peroxidase (APX), as well as non-enzymatic antioxidant defenses including phenolics and flavonoids (Rao et al. 2025). Consequently, the biochemical responses observed in pollen grains and pollen tubes under environmental stress conditions provide important indicators for evaluating the effects of stress (Mishra et al. 2023).

The responses of pollen tubes to environmental stress are also manifested at the molecular level. Although the functions of many stress-related proteins have been extensively studied in vegetative plant tissues, information regarding their roles in pollen under environmental stress conditions remains limited. Among the key components of these responses, heat shock proteins (HSPs) prevent protein denaturation under stress conditions and ensure proper protein folding and the maintenance of cellular homeostasis (Ren et al. 2019; Mukhopadhyay et al. 2024). Induced by nearly all environmental stress factors, HSPs are therefore widely used as biomarkers for evaluating plant stress responses (Pan et al. 2024). In pollen tubes, HSP70 is present in both cytosolic and membrane-associated fractions; while cytosolic HSP70 maintains protein homeostasis, membrane-associated HSP70 has been reported to contribute to the preservation of membrane integrity and fluidity, particularly under oxidative stress conditions (Parrotta et al. 2016; Ren et al. 2019). Although the presence of HSPs in pollen has been reported in studies such as Ren et al. (2019), these investigations have primarily focused on pollen preservation and cryopreservation rather than environmental stress responses during pollen tube growth. Another important protein involved in pollen tube stress responses is sucrose synthase (SuSy), which plays a critical role in pollen tube elongation by controlling the conversion of sucrose into UDP-glucose, a precursor for cell wall components, thereby influencing actin cytoskeleton organization and cell wall dynamics (Sedbrook 2004). Under many abiotic stress conditions, both the abundance and activity of SuSy change rapidly, and it is therefore considered a stress-sensitive protein (Conti et al. 2022). Its cytosolic form supports energy metabolism, whereas the membrane-associated form contributes to the supply of precursors required for cellulose and callose synthesis (Wang et al. 2014; Parrotta et al. 2016). However, compared with vegetative tissues, information regarding how SuSy responds to abiotic stress conditions in pollen remains relatively limited. Consequently, the combined evaluation of changes in HSP70 and SuSy levels provides valuable insights into the molecular responses of pollen tubes to environmental stresses.

However, despite the increasing number of studies addressing pollen responses to environmental stresses, the integrated mechanisms by which different levels of low-temperature stress influence pollen tube growth remain insufficiently understood. In particular, how structural changes in the pollen tube cell wall interact with antioxidant defense systems and stress-related proteins under varying temperature severity has not yet been comprehensively evaluated. The hypothesis of this study is that low temperature conditions may induce distinct regulatory adjustments in cytological, biochemical, and molecular parameters associated with pollen germination and pollen tube elongation in *Camellia sinensis*. In this context, pollen tube wall components, antioxidant systems reflecting oxidative balance, and stress-related proteins were considered as informative indicators for elucidating low temperature responses. Accordingly, the present study aimed to comparatively evaluate the responses of *C. sinensis* pollen exposed to different low temperature conditions (15, 10, and 5 °C) using explanatory parameters such as pollen germination, pollen tube elongation, pollen tube wall composition, oxidative balance, and stress-sensitive proteins. This integrative approach enables the responses of *C. sinensis* pollen to low temperature stress to be examined within a multidimensional framework, thereby contributing to a more comprehensive understanding of the sensitivity of generative processes to environmental stresses.

## Materials and methods

### Collection of pollen material

Pollen grains of *C. sinensis* (L.) O. Kuntze var. sinensis were collected from Çayeli district, Rize province, located in the Eastern Black Sea Region of Türkiye (41°06′57.0″ N, 40°45′27.0″ E) between September and October 2024. Pollen grains were collected from approximately 50 individual tea plants within the sampling area. Anthers were carefully removed from flowers using clean forceps and placed on clean white paper. The anthers were kept under yellow light overnight to allow pollen grains to be naturally released onto the paper surface. The released pollen grains were subsequently passed through pollen sieves to remove anther and filament debris and transferred into clean tubes. The pollen grains were dehydrated overnight in a desiccator containing silica gel and subsequently stored at -20 °C until use. Pollen viability was periodically monitored during the study using fluorescein diacetate/propidium iodide staining to confirm that the stored pollen remained viable throughout the experiments (Novara et al. 2017). To minimize plant-to-plant variation and ensure sample uniformity, pollen samples collected from different plants were pooled and thoroughly mixed after dehydration to create a homogeneous pollen batch used for all subsequent experiments. Therefore, the pooled pollen sample was considered as a single biological replicate in this study. All subsequent measurements reflect technical variation, variation among individual pollen grains within the same biological sample, and responses to experimental treatments rather than variation between independent biological replicates.

## In vitro pollen germination and basic pollen analyses

For in vitro pollen germination assays, a modified Brewbaker and Kwack (B&K) (1963) germination medium supplemented with 8% sucrose was used. Pollen grains in the control group were germinated at 20 °C, whereas pollen grains in the treatment groups were germinated at 15, 10, and 5 °C for 4 h. The 4 h incubation period was selected to allow sufficient pollen tube growth for reliable measurements and fluorescence analyses while avoiding excessive tube elongation. The control temperature (20 °C) was selected because tea pollination generally occurs within the optimal temperature range of approximately 20–27 °C (Harbowy et al. 1997; Duncan et al. 2016; Hajiboland 2017), and the lower temperatures were used to establish a gradient of increasing low-temperature stress. The low-temperature treatments were carried out using a temperature-controlled laboratory refrigerator (YUI, China). The temperature settings were calibrated before each experimental set and periodically monitored during the 4 h germination period to ensure temperature stability. Pollen grains were observed using an Olympus BX51 light microscope. Pollen germination was defined as the condition in which the pollen tube length exceeded the diameter of the pollen grain. Pollen germination rates and pollen tube lengths were calculated using ImageJ software. Pollen tube length was measured only in germinated pollen grains. Non-germinated pollen grains were not included in the measurements and were not assigned a value of zero. For each treatment group, three independent microscope slides were prepared as technical replicates, and at least 150 randomly selected pollen grains and pollen tubes were analyzed per slide to account for variation among individual pollen grains within the same biological sample.

## Analysis of pollen tube cell wall components

Germinated pollen grains were stained with Calcofluor White (CFW) for cellulose (Peng et al. 2021), Aniline Blue (AB) for callose (Bougourd et al. 2000), and propidium iodide (PI) for newly secreted cell wall material. These dyes were applied directly to the pollen tubes, and staining was performed without fixation to allow visualization of living pollen tubes. To examine the distribution of methyl-esterified and de-esterified acidic pectins, germinated pollen grains were fixed in PEM buffer (0.5 M PIPES pH 6.9, 100 mM EGTA pH 7, 100 mM MgCl₂) containing 3% paraformaldehyde for 30 min at room temperature in the dark. After fixation, pollen grains were washed 2 times with PM buffer by centrifuging at 2500 g for 8 min. For the detection of de-esterified acidic and methyl-esterified pectins, pollen grains were incubated overnight at 4 °C with primary antibodies JIM5 (1:20) (Agrisera) and JIM7 (1:10) (Agrisera), respectively (Li et al. 1994). Subsequently, pollen grains were washed 2 times with PM buffer by centrifuging at 2500 g for 8 min and samples were incubated with Alexa Fluor 488 goat anti-rat IgG secondary antibody (1:50) (Invitrogen) for 45 min at 37 °C (Biagini et al. 2014). After washing 2 times in PM buffer by centrifuging at 2500 g for 8 min, pollen grains were examined using an Olympus BX51 fluorescence microscope, and images were captured using the KAMERAM software. During observation and image acquisition, excitation/emission wavelength ranges of 365–432 nm for CFW, 455–495 nm for AB, 482–608 nm for PI, and 488–522 nm for methyl-esterified and de-esterified acidic pectins were used. Care was taken to apply comparable focal planes and identical illumination settings for each fluorescent dye and antibody during imaging. For quantitative analysis, three independent microscope slides were prepared for each treatment group group as technical replicates, and a total of at least 20 pollen tubes of comparable length were analyzed per group to capture variation among individual pollen tubes within the same biological sample. To evaluate fluorescence accumulation at the pollen tube apex, normalized fluorescence intensity was quantified within a defined 100 μm² area of the apical region using the ‘Rectangle Selection’ tool in ImageJ. Background fluorescence was subtracted to eliminate noise. The selected regions of interest used for these measurements are indicated in the corresponding figures. In addition, fluorescence distribution along the pollen tube was analysed by tracing the tube axis from the apical tip up to 50 μm towards the base using the ‘Segmented Line’ tool in ImageJ with an approximate line width of 1 μm. The analysed segments are illustrated in the figures. Representative fluorescence profiles were generated based on the average of 20 independent measurements.

## Analysis of enzymatic antioxidant activities

For the determination of SOD, CAT, and APX activities, 50 mg of pollen grains were germinated for each group. Homogenization buffers consisting of 50 mM PBS (pH 7.8) containing 0.5 M EDTA and 4% PVP were used for SOD and CAT analyses, whereas a buffer containing 0.5 M EDTA, 2% PVP, and 20 mM ascorbic acid was used for APX analysis. The homogenates were centrifuged at 12.000 g for 15 min, and the resulting supernatants were used for enzymatic analyses (Giannopolitis and Ries 1977; Aebi 1984; Nakano and Asada 1981). Protein concentrations of the homogenates were determined using the Bradford method (Bradford 1976) with bovine serum albumin as the standard (Supplementary Table 1). SOD activity was determined according to the riboflavin-NBT method. For this purpose, the supernatant was mixed with SOD assay buffer (100 mM PBS pH 7, 2 M Na₂CO₃, 0.5 M EDTA, 130 mM L-methionine, 750 µM NBT) and riboflavin, and the samples were incubated under white light at an intensity of 50 µmol m⁻² s⁻¹ for 2 min. At the end of the reaction, absorbance was measured spectrophotometrically at 560 nm, and a negative control was used as a blank (Çakmak and Marschner 1992). CAT activity was determined by monitoring the decrease in absorbance at 240 nm associated with the decomposition of H₂O₂. The CAT assay buffer (200 mM PBS pH 7.0, 72 mM H₂O₂) was pre-incubated at 30 °C for 3 min, after which the supernatant was added, and changes in absorbance were recorded kinetically for 2 min (Prochazkova et al. 2001). APX activity was determined using a method based on ascorbate oxidation. The APX assay buffer (50 mM PBS pH 7, 5 mM ascorbate, 1 mM EDTA, 1 mM H₂O₂) was mixed with the supernatant, and the reaction was monitored kinetically at 290 nm for 2 min (Liu et al. 2008). In all enzymatic analyses, the corresponding assay buffers were used as blanks. Enzyme activities were expressed per mg of total protein to normalize the measurements among samples. For each treatment group, a single pooled pollen extract was used, and all spectrophotometric measurements were performed in triplicate as technical replicates to ensure measurement reliability. Therefore, the observed variation reflects technical variation rather than differences between independent biological replicates.

## Analysis of antioxidant capacity and non-enzymatic antioxidant compounds

For each group, 50 mg of pollen grains were germinated and homogenized in 100% ethanol by ultrasonication for 30 min. The homogenates were centrifuged at 10.000 g for 15 min, and the resulting supernatants were used for antioxidant analyses. For each treatment condition, a single extract was prepared. Total antioxidant activity was determined using the 2,2-diphenyl-1-picrylhydrazyl (DPPH) radical scavenging assay (Chatatikun and Chiabchalard 2013). For this purpose, 50 µl of supernatant was mixed with 100 µl of DPPH solution and incubated for 30 min at room temperature in the dark. Absorbance values were measured at 517 nm using a Cytation-3 (Biotek) microplate reader. L-ascorbic acid was used as a positive control, and 100% ethanol served as the blank. Total phenolic content was determined using the Folin-Ciocalteu method (Clarke et al. 2013). In this assay, 40 µl of supernatant was mixed with 100 µl of Folin-Ciocalteu reagent and 75 µl of 7.5% Na₂CO₃, followed by incubation in the dark for 60 min. Absorbance was measured at 750 nm, and the results were calculated as gallic acid equivalents. Total flavonoid content was determined using the aluminum chloride method (Yang et al. 2011). For this purpose, 75 µl of supernatant was mixed with 75 µl of 2% AlCl₃ and 75 µl of sodium acetate. After 15 min of incubation, absorbance was measured at 435 nm, and the results were expressed as rutin equivalents. In all analyses, 100% ethanol was used as the blank. For each treatment condition, a single pooled extract was prepared, and each spectrophotometric measurement was performed in triplicate as technical replicates. Therefore, the observed variation reflects measurement variability rather than differences between independent biological replicates.

### Analysis of stress-related proteins

For each group, 50 mg of pollen grains were germinated and homogenized in HEM buffer (100 mM HEPES pH 7.5, 50 mM EGTA, 50 mM MgCl₂) containing 1 mM DTT using a Retsch Vibromill homogenizer at 30 Hz for 4 min. The homogenates were centrifuged sequentially at 500 g (4 °C, 10 min) and 16,000 g (4 °C, 60 min); the resulting pellet was used as the membrane fraction, whereas the supernatant was collected as the cytoplasmic fraction (Del Casino et al. 2024). Cytosolic and membrane protein fractions were treated as independent extracts for subsequent analyses. Stress-related proteins were analyzed by one-dimensional SDS-PAGE followed by immunoblotting. Protein concentrations of cytosolic and membrane extracts were determined using a protein quantification kit (Takara Bio, Japan) (Supplementary Table 2). Equal amounts of protein (10 µg per lane) were loaded onto SDS-PAGE gels for immunoblot analysis. Electrophoresis was performed using 10% TGX Stain-Free gels at 200 V for approximately 35 min, and proteins were transferred onto nitrocellulose membranes using a Trans-Blot Turbo system. The Stain-Free technology was used to visualize total protein signals and to allow normalization among electrophoretic lanes. Membranes were incubated with primary antibodies against HSP70 (rabbit polyclonal,1:5000; Agrisera) (Piccini et al. 2021) and SuSy1 (rabbit polyclonal, 1:5000; Agrisera) for 1 h at room temperature (Persia et al. 2008), followed by incubation with an Alexa Fluor 594-conjugated secondary antibody (1:50) for 1 h at room temperature. Immunoblot signals were quantified using Image Lab software (v6.1). Total protein signals were visualized in the Stain-Free Blot channel, and the lane exhibiting the highest total protein signal was used as the reference for normalization. The protein content of all other lanes on the same membrane was adjusted relative to this reference lane. Subsequently, immunoblot band intensities were normalized based on the corresponding total protein signal in each lane. The obtained I.D. values were normalized separately for each treatment and fraction on a scale from 1 to 100 to facilitate comparison among treatments. Immunoblot analyses were performed using single cytosolic and membrane extracts per treatment condition derived from pooled pollen samples. Therefore, the results represent treatment-dependent changes rather than variation between independent biological replicates.

## Statistical analyses and heat map analysis

Statistical comparisons of measurement results were performed using one-way ANOVA followed by Tukey’s HSD post hoc test in the SPSS software (*p* < 0.05) (Koubouris et al. 2009). Statistical analyses were performed using the mean values obtained from replicate measurements for each treatment group, including technical replicates and multiple measurements across individual pollen grains/tubes within the same pooled biological sample. In the heat map analysis, the control group was used as the reference, and the effects of low temperature treatments were calculated as percentage changes of each parameter relative to the control using the formula: [(Treatment − Control) / Control] × 100. For parameters showing a spatial distribution along the pollen tube (CFW, AB, PI, JIM7, and JIM5), the similarity between treatment and control profiles was evaluated by regression analysis using the coefficient of determination (R²). To quantify the magnitude of deviation from the control profile, the transformation (1 − R²) was applied. This approach was preferred because R² reflects the degree of similarity rather than directional change; therefore, (1 − R²) provides a direct measure of dissimilarity independent of whether the deviation corresponds to an increase or decrease. Because the direction of change (increase or decrease) was not considered in the integrative visualization, absolute values were used to represent the magnitude of deviation from the control.To enable integrated evaluation of all parameters, a heat map was generated in the RStudio environment using the pheatmap function, with row scaling applied and values normalized to a 1–7 range solely for visualization and comparative representation (Galili et al. 2018). The resulting scores should therefore be interpreted as relative indicators of treatment-induced changes rather than absolute quantitative measurements of the analyzed parameters. All measured parameters in the study were included in the heatmap analysis without prior selection in order to provide an integrated visualization of the overall response to low-temperature stress.

## Results

### Basic pollen analyses

**Fig. 1:**
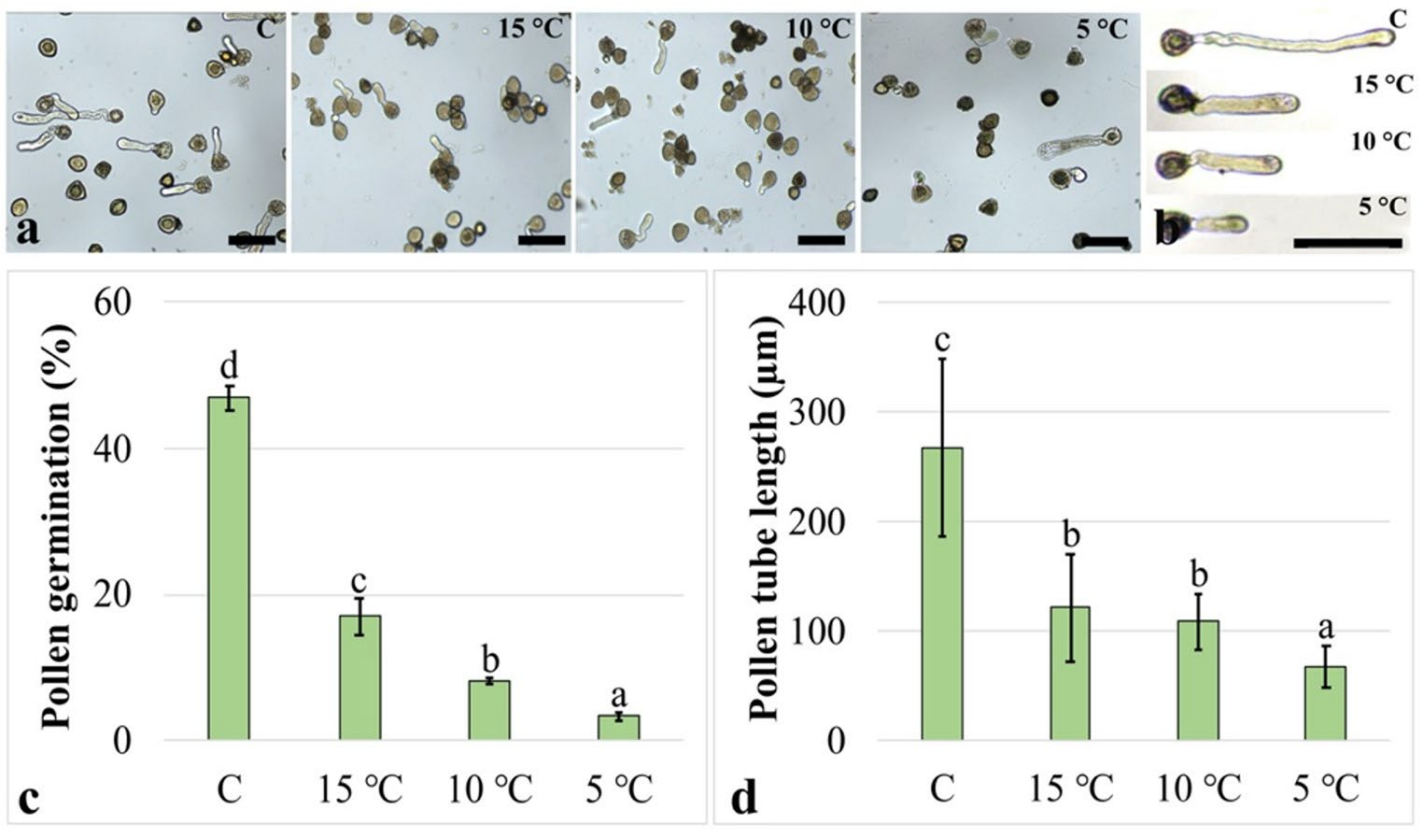
Effects of low-temperature stress on basic pollen parameters. **(a)** Representative micrographs of pollen germination. **(b)** Representative micrographs of pollen tube. **(c)** Pollen germination rate. **(d)** Pollen tube length. Bar: 150 μm. Images are representative micrographs selected from multiple preparations and microscopic fields, while quantitative data shown in the graphs are based on measurements from numerous pollen grains. Distinct letters point out the statistically significant differences (*P* < 0.05) and error bars indicate the standard deviations

To evaluate the effects of low temperature stress on basic pollen parameters, pollen germination rates and pollen tube lengths were determined. Representative micrographs are presented in Fig. 1a, b. In parallel with the decrease in treatment temperature, pollen germination rates were markedly reduced in all treatment groups compared with the control group. The reduction was determined to be 63.7% after the 15 °C treatment, 82.6% after the 10 °C treatment, and 92.9% after the 5 °C treatment. In addition, statistically significant differences were detected among the treatment groups (Fig. 1c). Similarly, pollen tube lengths showed a pronounced decrease in all treatment groups relative to the control as the treatment temperature decreased. The reduction in pollen tube length was 54.4% after the 15 °C treatment, 59.2% after the 10 °C treatment, and 74.4% after the 5 °C treatment. However, no significant difference was detected between the 15 °C and 10 °C treatment groups (Fig. 1d). Pollen tubes maintained normal morphology, with no apparent tip swelling or bursting under low-temperature treatments.

### Analysis of pollen tube cell wall components

**Fig. 2:**
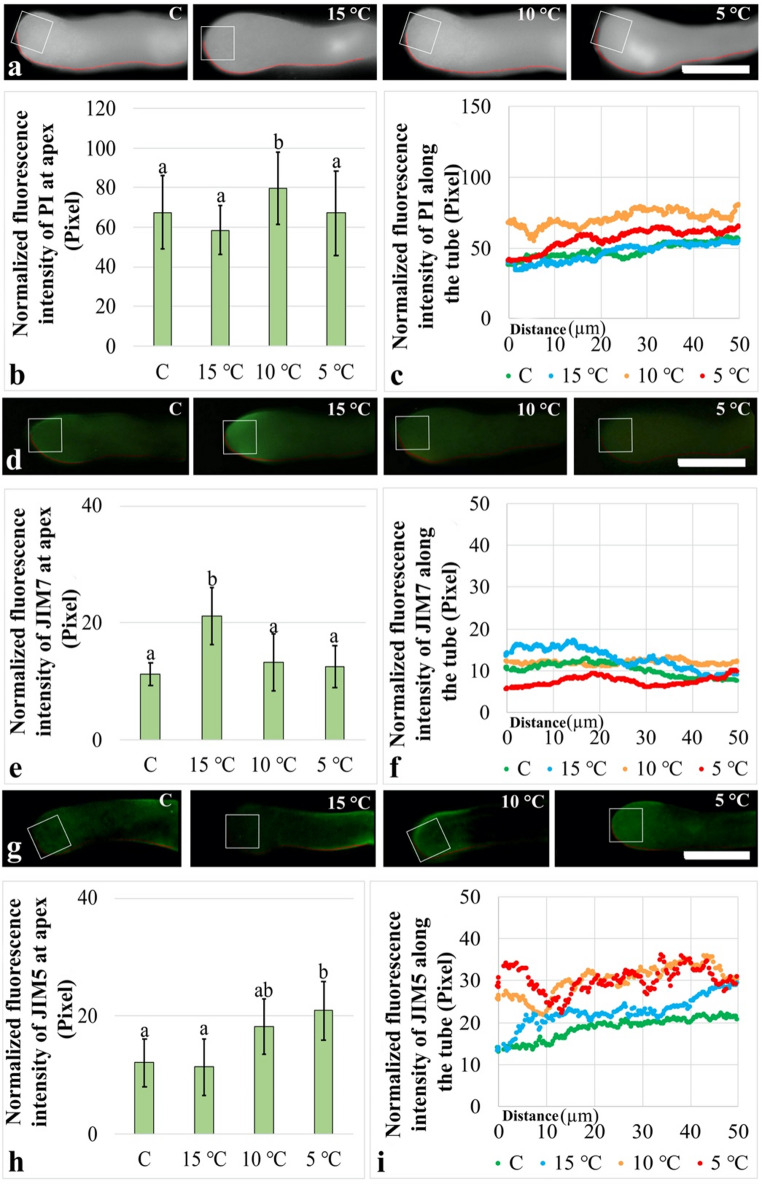
Effects of low-temperature stress on the distribution of newly synthesized cell wall material, methyl-esterified, and de-esterified acidic pectins in pollen tubes. **(a)** Representative micrographs showing PI labelled newly secreted cell wall material distribution. **(b)** PI fluorescence intensity within a 100 μm² apical area (indicated as box). **(c)** PI fluorescence intensity within a 50 μm cell wall segment (indicated as red line). **(d)** Representative micrographs showing JIM7 labelled methyl-esterified pectin distribution. **(e)** JIM7 fluorescence intensity within a 100 μm² apical area. **(f)** JIM7 fluorescence intensity within a 50 μm cell wall segment. **(g)** Representative micrographs showing JIM5 labelled de-esterified acidic pectin distribution. **(h)** JIM5 fluorescence intensity within a 100 μm² apical area. **(i)** JIM5 fluorescence intensity within a 50 μm cell wall segment. Bar: 20 μm. Fluorescence intensity measurements were obtained from 20 independent pollen tubes for each treatment group (*n* = 20). The images presented are representative examples of the observed experimental results. Distinct letters point out the statistically significant differences (*P* < 0.05) and error bars indicate the standard deviations

To investigate the effects of low temperature stress on pollen tube cell wall components, the distribution of newly secreted cell wall material in pollen tubes was first examined using PI staining. In pollen tubes from both control and treatment groups, newly secreted cell wall material were predominantly distributed in the apical and neck regions (Fig. 2a). For more detailed analysis, PI fluorescence intensity was measured within a defined apical area of 100 μm². At the apex, PI fluorescence intensity showed a significant increase of 17.7% compared with the control only after the 10 °C treatment, whereas no significant differences were detected in the other treatment groups relative to the control. In addition, no significant difference was observed between the 15 °C and 5 °C treatments (Fig. 2b). For a more comprehensive evaluation, PI fluorescence intensity was measured along a 50 μm cell wall segment extending from the apical region toward the neck region. The results revealed that the intensity curves of the control and 15 °C groups exhibited similar profiles, with PI fluorescence intensity showing a slight increase from the apex toward the neck region. Although numerical differences relative to the control were observed after the 5 °C treatment, the overall profile of the intensity curve was found to be highly similar to that of the control group. In contrast, pronounced deviations from the control profile were detected in the intensity curve following the 10 °C treatment (Fig. 2c). Regression analyses performed to evaluate the similarity of the curves obtained from the treatment groups to that of the control group further supported these findings. Accordingly, R² values were calculated as 0.62 for the 15 °C group, 0.002 for the 10 °C group, and 0.53 for the 5 °C group.

To obtain more sensitive data, the distribution of methyl-esterified pectins in pollen tubes was examined using JIM7 immunolabeling. In pollen tubes from the control and 15 °C groups, the JIM7 signal was more prominent in the apical region. In contrast, a relative reduction in JIM7 signal intensity was observed following the 10 °C and 5 °C treatments (Fig. 2d). For detailed analysis, JIM7 fluorescence intensity was measured within a defined apical area of 100 μm². At the apex, JIM7 fluorescence intensity showed a significant increase of 88.2% compared with the control only after the 15 °C treatment, whereas no significant differences relative to the control were detected in the other treatment groups. Moreover, no significant difference was observed between the 10 °C and 5 °C treatments (Fig. 2e). For a more comprehensive evaluation, JIM7 fluorescence intensity was measured along a 50 μm cell wall segment extending from the apical region toward the neck region. Although numerical differences were observed between the control and 15 °C groups, the overall trend indicated a decrease in JIM7 fluorescence intensity from the apex toward the neck region. Following the 10 °C treatment, the intensity profile became uniform along the analyzed segment. Similarly, after the 5 °C treatment, JIM7 fluorescence intensity remained low and exhibited an almost uniform profile, except for a slight increase within the 10–30 μm segment (Fig. 2f). Consistent with these observations, R² values were calculated as 0.63 for the 15 °C group, 0.07 for the 10 °C group, and 0.007 for the 5 °C group.

To achieve a clearer assessment, the distribution of de-esterified acidic pectins in pollen tubes was examined using JIM5 immunolabeling. In pollen tubes from the control and 15 °C groups, the JIM5 signal was more pronounced in the neck region, whereas following the 10 °C and 5 °C treatments, the signal was found to be more intense in the apical region (Fig. 2g). For detailed analysis, JIM5 fluorescence intensity was measured within a defined apical area of 100 μm². At the apex, JIM5 fluorescence intensity showed a significant increase of 73.2% compared with the control only after the 5 °C treatment, whereas no significant differences relative to the control were detected in the other treatment groups. In addition, no significant difference was observed between the 10 °C group and the other treatment groups, while a statistically significant difference was detected between the 15 °C and 5 °C treatments (Fig. 2h). For a more comprehensive evaluation, JIM5 fluorescence intensity was measured along a 50 μm cell wall segment extending from the apical region toward the neck region. In the control and 15 °C groups, the overall trend was highly similar, with JIM5 fluorescence intensity increasing from the apex toward the neck region. In contrast, marked deviations from the control profile were observed in the 10 °C and 5 °C treatment groups (Fig. 2i). Consistent with these observations, R² values were calculated as 0.65 for the 15 °C group, 0.73 for the 10 °C group, and 0.053 for the 5 °C group.

**Fig. 3:**
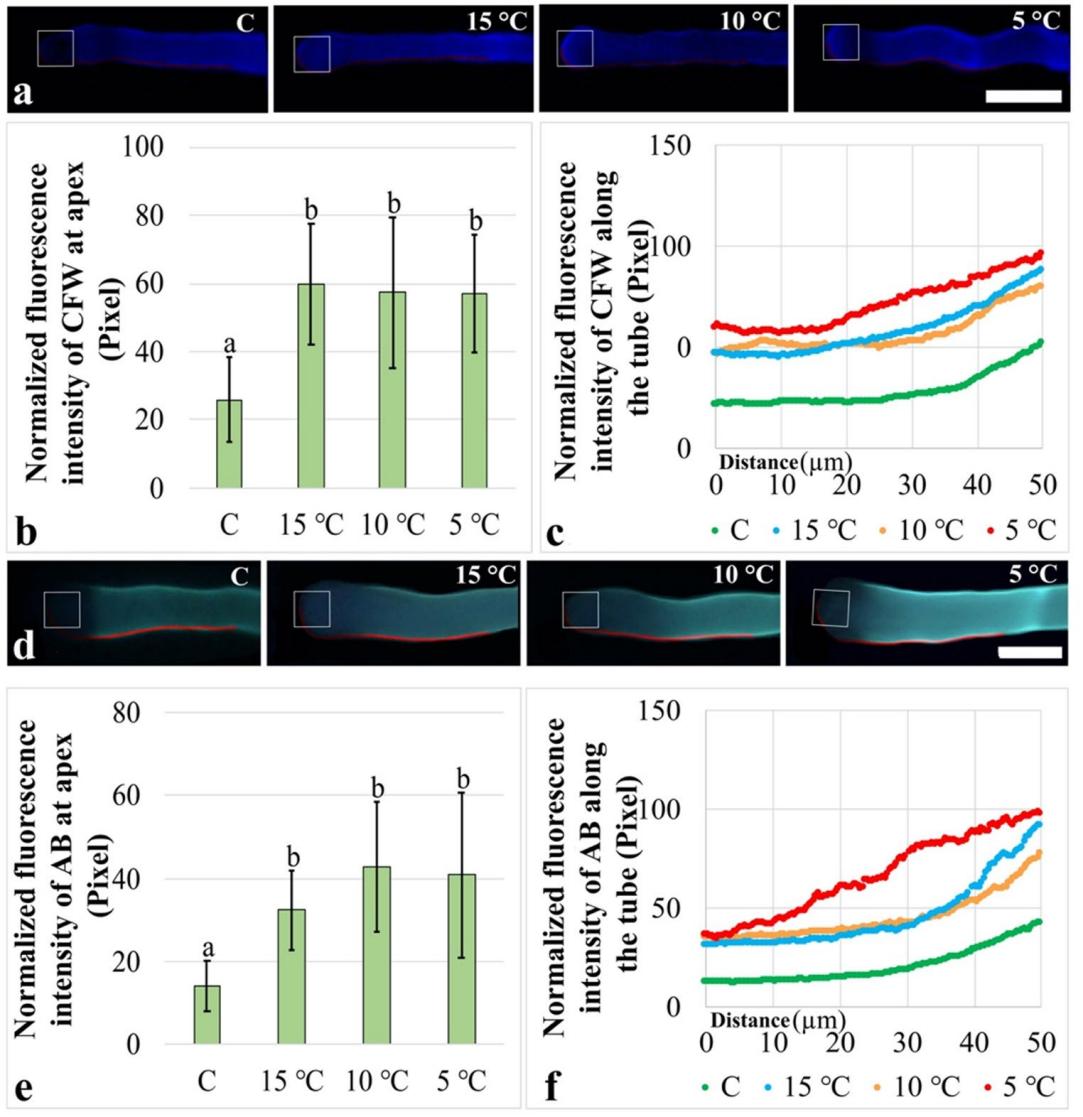
Effects of low-temperature stress on the distribution of cellulose and callose in pollen tubes. **(a)** Representative micrographs showing CFW labelled cellulose distribution. **(b)** CFW fluorescence intensity within a 100 μm² apical area (indicated as box). **(c)** CFW fluorescence intensity within a 50 μm cell wall segment. **(d)** Representative micrographs showing AB labelled callose distribution. **(e)** AB fluorescence intensity within a 100 μm² apical area. **(f)** AB fluorescence intensity within a 50 μm cell wall segment. Bar: 20 μm. luorescence intensity measurements were obtained from 20 independent pollen tubes for each treatment group (*n* = 20). The images presented are representative examples of the observed experimental results. Distinct letters point out the statistically significant differences (*P* < 0.05) and error bars indicate the standard deviations

Cellulose distribution in pollen tubes was analyzed using CFW staining. In pollen tubes from both the control and treatment groups, cellulose distribution was clearly observed in both the apical and neck regions (Fig. 3a). For detailed analysis, CFW fluorescence intensity was measured within a defined apical area of 100 μm². At the apex, CFW fluorescence intensity showed a significant increase in all treatment groups compared with the control. The increase was determined to be 130.5% after the 15 °C treatment, 121.4% after the 10 °C treatment, and 120.0% after the 5 °C treatment. However, no significant differences were detected among the treatment groups (Fig. 3b). For a more comprehensive evaluation, CFW fluorescence intensity was measured along a 50 μm cell wall segment extending from the apex toward the neck region, representing a spatial distribution profile rather than a single apical measurement. The control and treatment groups exhibited similar slope trends, with fluorescence intensity increasing from the apex toward the neck region. Notably, the lowest numerical values were observed in the control group, whereas the highest values were detected in the 5 °C group (Fig. 3c). Consistent with these observations, R² values were calculated as 0.94 for the 15 °C group, 0.97 for the 10 °C group, and 0.81 for the 5 °C group.

Finally, callose distribution in pollen tubes was analyzed using AB staining. In pollen tubes from both the control and treatment groups, callose accumulation was more prominent in the neck region (Fig. 3d). For detailed analysis, AB fluorescence intensity was measured within a defined apical area of 100 μm². At the apex, AB fluorescence intensity showed a significant increase in all treatment groups compared with the control. The increase was determined to be 129.7% after the 15 °C treatment, 203.9% after the 10 °C treatment, and 189.7% after the 5 °C treatment. However, no significant differences were detected among the treatment groups (Fig. 3e). For a more comprehensive evaluation, AB fluorescence intensity was measured along a 50 μm cell wall segment. The control, 15 °C, and 10 °C groups exhibited similar trend profiles, with fluorescence intensity increasing from the apex toward the neck region. Although a similar increase from the apex to the neck region was also observed in the 5 °C group, its slope profile differed from those of the other groups. In addition, the lowest numerical values were observed in the control group, whereas the highest values were detected in the 5 °C group (Fig. 3f). Consistent with these observations, R² values were calculated as 0.01 for the 15 °C group, 0.98 for the 10 °C group, and 0.001 for the 5 °C group.

### Analysis of enzymatic antioxidant activities

**Fig. 4:**
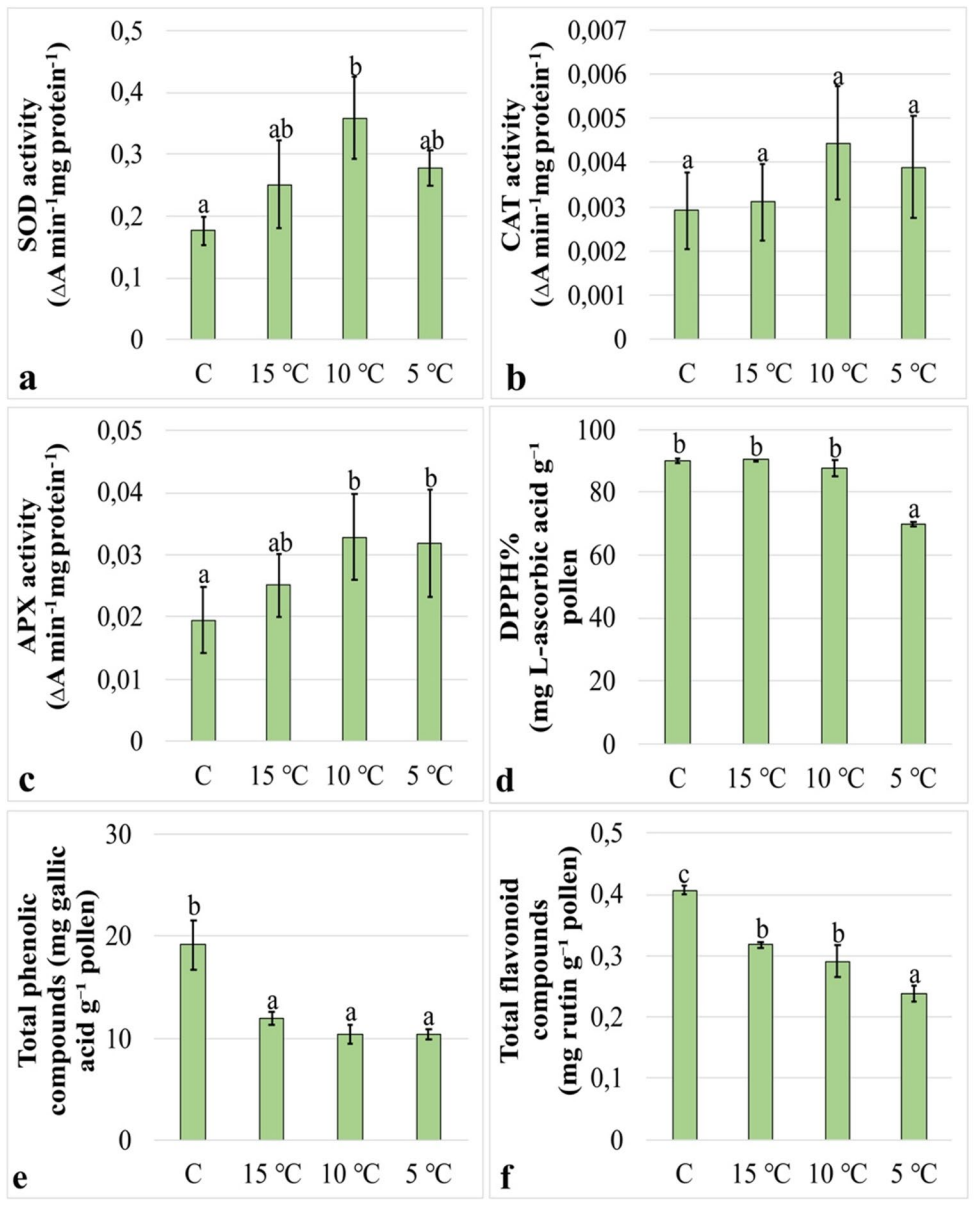
Effects of low-temperature stress on enzymatic and non-enzymatic antioxidant systems. **(a)** SOD activity. **(b)** CAT activity. **(c)** APX activity. **(d)** %DPPH activity. **(e)** Total phenolic content. **(f)** Total flavonoid content. Distinct letters point out the statistically significant differences (*P* < 0.05) and error bars indicate the standard deviations

To evaluate enzymatic antioxidant defense responses in pollen grains under low temperature stress, the activities of SOD, CAT, and APX were analyzed. SOD activity showed a statistically significant increase of 111.0% compared with the control only after the 10 °C treatment, whereas no significant differences relative to the control were detected in the other treatment groups. In addition, no significant differences were observed among the treatment groups (Fig. 4a). No significant differences in CAT activity were detected between the control and treatment groups (Fig. 4b). In contrast, APX activity increased significantly compared with the control by 73.0% after the 10 °C treatment and by 67.7% after the 5 °C treatment, while no significant change relative to the control was observed in the 15 °C group. Furthermore, no significant differences were detected among the treatment groups (Fig. 4c).

### Analysis of antioxidant capacity and non-enzymatic antioxidant compounds

To assess non-enzymatic antioxidant defense responses in pollen tubes under low temperature stress, %DPPH activity together with total phenolic and flavonoid contents were analyzed. %DPPH activity showed a statistically significant decrease of 22.2% compared with the control only after the 5 °C treatment, whereas no significant differences relative to the control were detected in the other treatment groups. No significant difference in %DPPH activity was observed between the 15 °C and 10 °C groups; however, both groups differed significantly from the 5 °C group (Fig. 4d). Total phenolic content was significantly reduced in all treatment groups compared with the control. The reduction was determined to be 37.5% after the 15 °C treatment, 46.0% after the 10 °C treatment, and 45.6% after the 5 °C treatment. No significant differences were detected among the treatment groups (Fig. 4e). Similarly, total flavonoid content was significantly decreased in all treatment groups relative to the control. The reduction was 20.6% after the 15 °C treatment, 27.1% after the 10 °C treatment, and 40.5% after the 5 °C treatment. No significant difference was observed between the 15 °C and 10 °C groups; however, both groups differed significantly from the 5 °C group (Fig. 4f).

### Analysis of stress-related proteins

**Fig. 5:**
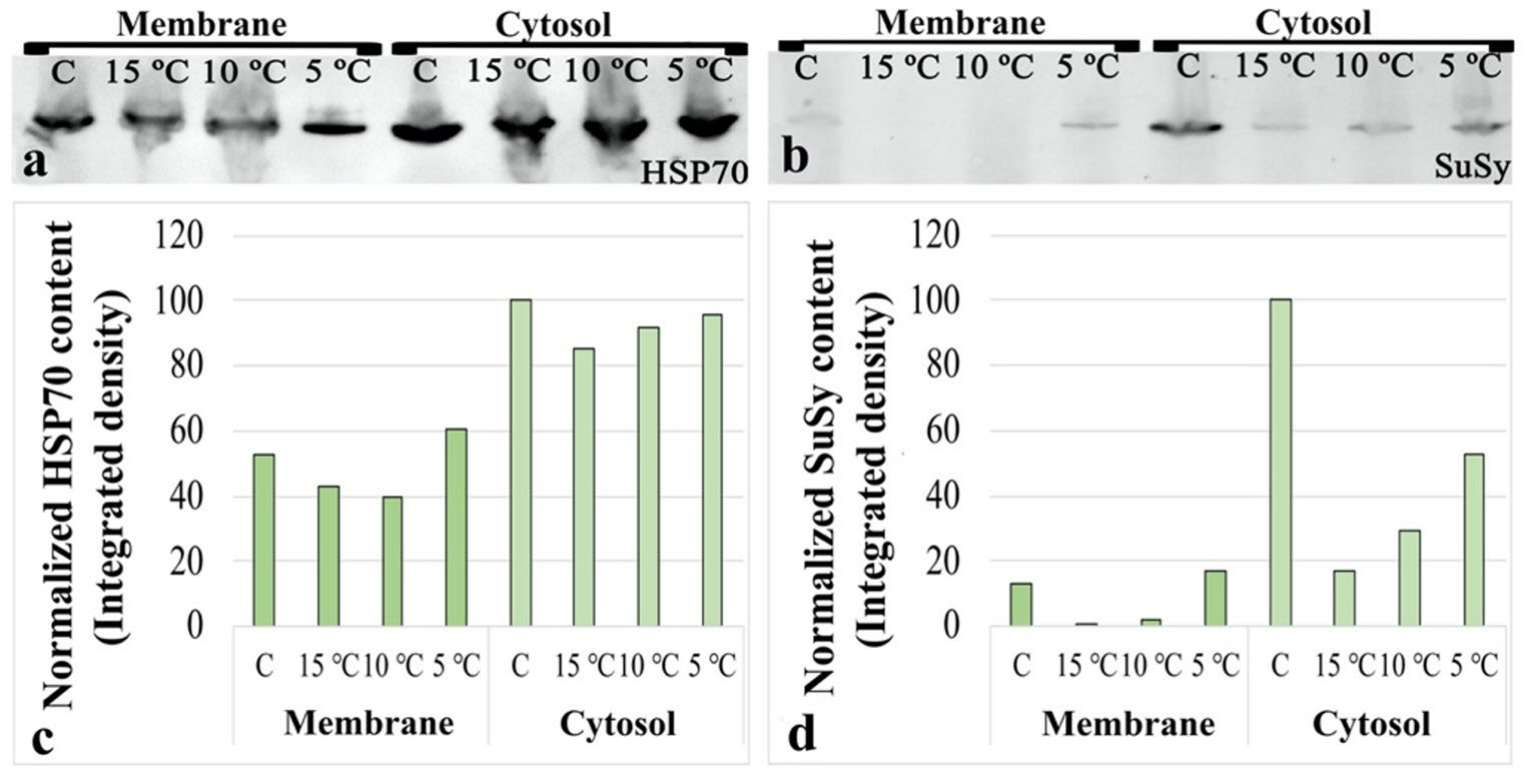
Effects of low-temperature stress on stress-related protein levels. **(a)** Representative raw immunoblot results for HSP70. **(b)** Representative raw immunoblot results for SuSy **(c)** HSP70 band intensity. **(d)** SuSy band intensity

To further evaluate the effects of low temperature stress, protein levels of HSP70 and SuSy were analyzed separately in cytosolic and membrane-associated fractions to evaluate their compartment-specific responses to low temperature stress. Stain-Free gel images showing total protein signals are provided in Supplementary Figure. Immunoblotting data revealed that both target proteins were present in all groups in both membrane and cytosolic fractions, although their distribution patterns varied. HSP70 was slightly more abundant in the cytosolic fraction but overall exhibited a relatively balanced distribution between the two fractions (Fig. 5a). In contrast, SuSy was more abundant in the cytosolic fraction, while showing a weaker signal in the membrane fraction (Fig. 5b). In the membrane fraction, HSP70 levels decreased in the 15 °C and 10 °C groups compared with the control, whereas an increase relative to the control was observed in the 5 °C group. In the cytosolic fraction, HSP70 levels decreased in all treatment groups compared with the control; however, the highest value among the treatment groups was detected in the 5 °C group (Fig. 5c). Similarly, in the membrane fraction, SuSy levels decreased in the 15 °C and 10 °C groups relative to the control, while an increase compared with the control was observed in the 5 °C group. In the cytosolic fraction, SuSy levels were reduced in all treatment groups relative to the control, with the highest value among the treatment groups again detected in the 5 °C group (Fig. 5d).

### Heat map analysis

**Fig. 6:**
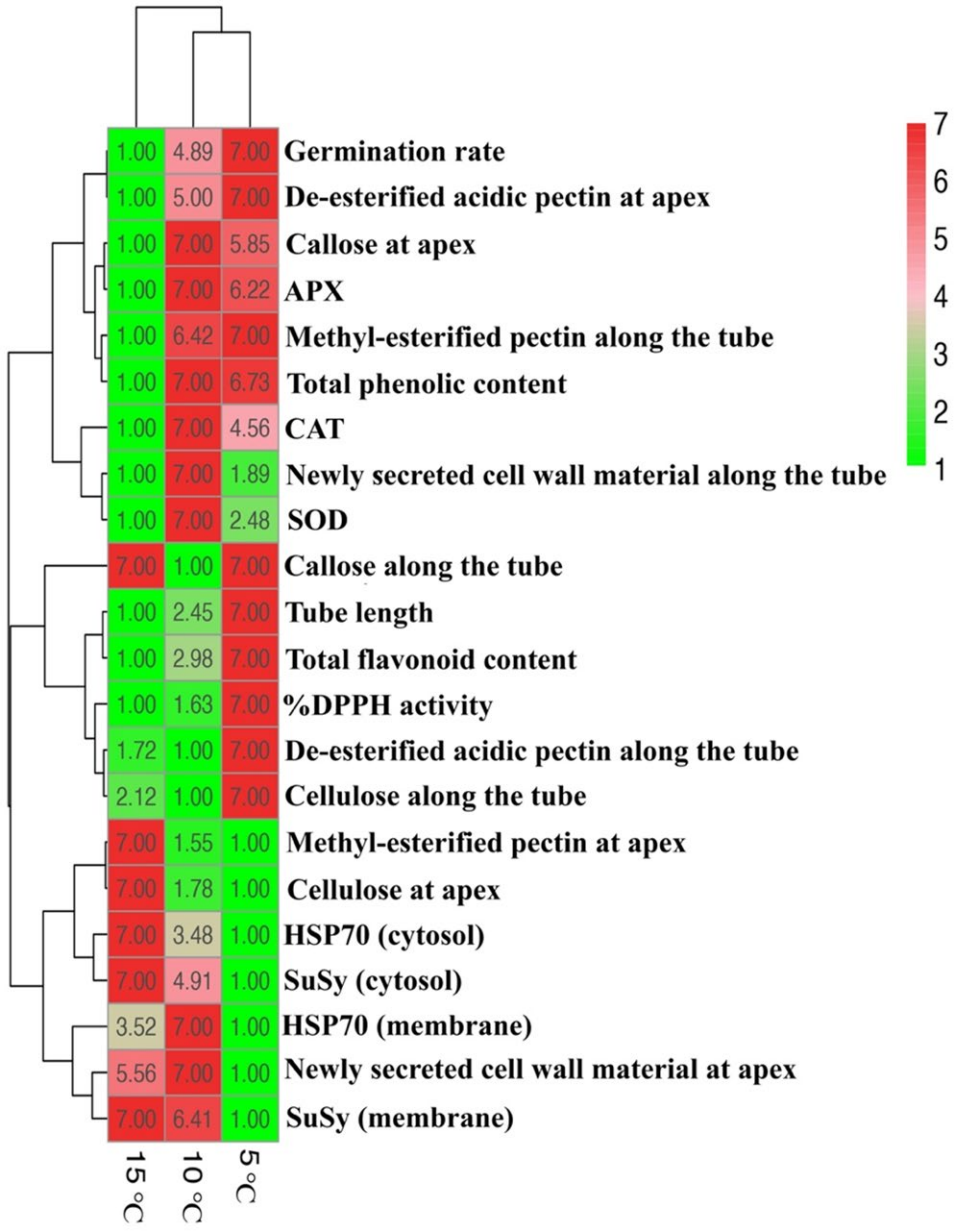
Heat map analysis of treatment-induced changes across measured parameters

To enable an integrated comparison of parameter changes across all treatment groups, a heat map analysis was performed. The control group was considered the optimal condition, and change values for each parameter in each treatment group were calculated relative to the control. As the effects of increases or decreases in the parameters on pollen tube performance were not known, absolute values of the changes were used for heat map construction. According to the heat map, groups exhibiting the highest magnitude of changes were represented by the most intense red color, whereas groups with the lowest magnitude of changes were represented by the most intense green color. In addition, the total values of the cells in each column were calculated to generate a ranking. Based on this ranking, the treatment groups were ordered from the highest to the lowest overall magnitude of change as follows: 5 °C (score: 195.46), 10 °C (score: 100.5), and 15 °C (score: 66.92). Furthermore, the heat map analysis indicated that the 10 °C and 5 °C groups clustered within the same subcluster, which subsequently merged with the 15 °C group at a higher hierarchical level (Fig. 6).

## Discussion

The effects of low temperature stress on basic pollen parameters have been reported in various plant species (Parrotta et al. 2019; Laggoun et al. 2021; Liu et al. 2023). Consistent with the literatures, our results demonstrated that exposure of *C. sinensis* pollen to 15, 10, and 5 °C significantly reduced both pollen germination rates and pollen tube lengths compared with the control. The effects of low temperature stress in *C. sinensis* have been extensively investigated in previous studies. It has been reported that a 2-h exposure to 4 °C reduced pollen germination by 54% and pollen tube elongation by 50% (Wang et al. 2012), whereas a 3-h exposure reduced these parameters by 10% and 16%, respectively (Çetinbaş-Genç et al. 2020). In the present study, however, a 4-h exposure to low temperature stress at 5 °C resulted in a 92% reduction in pollen germination and a 74% reduction in pollen tube length. Although all three studies were conducted using *C. sinensis* (L.) O. Kuntze pollen under comparable low temperature conditions, the observed differences may be attributed to methodological variables such as the physiological status of the pollen, collection time, environmental history, and differences in germination media. This interpretation is consistent with previous reports indicating that stress responses in pollen of the same species are shaped by multiple interacting factors (Brewbaker and Kwack 1963; Firon et al. 2012).

Environmental stress can modify the spatial organization of pollen tube wall components and thereby affect pollen tube elongation (Breygina et al. 2012; Fang et al. 2016). In agreement with these reports, our results show that low temperature altered the distribution of pollen tube wall components in *C. sinensis*, and these alterations were associated with reduced pollen tube length. Numerous studies have demonstrated that environmental stress factors modify pectin distribution within the pollen tube wall and that stress-induced decreases in pectin content affect pollen tube elongation (Rounds et al. 2014; Parrotta et al. 2016; Liu et al. 2021; Hou et al. 2021). In *Pyrus communis*, newly secreted cell wall materials were shown to be distributed along both the tip and the shank of the pollen tube, with the highest intensity localized at the apical region (Aloisi et al. 2017). In the present study, newly secreted cell wall material accumulated in both the apical and shank regions of pollen tubes in all groups; however, in contrast to the findings of Aloisi et al. (2017), a higher accumulation was observed in the neck region compared with the apex. This discrepancy may be attributed to species-specific cell wall organization strategies and to differences in pollen tube growth dynamics among species. In *Nicotiana tabacum*, newly secreted cell wall materials were reported to accumulate at the pollen tube tip under control conditions, and this apical accumulation was abolished following exposure to 4 °C (Parrotta et al. 2019). In contrast, in our study, a significant alteration in pectin distribution relative to the control was observed only after the 10 °C treatment, suggesting that pectin remodeling dynamics may be shaped in distinct ways depending on both the applied temperature regime and species-specific characteristics. This interpretation is consistent with previous studies indicating that the effects of environmental stresses on pollen tube wall components can vary among plant species (Fang et al. 2016).

In pollen tubes, methyl-esterified pectins are generally concentrated at the apical region, whereas de-esterified acidic pectins are distributed along the shank of the tube. However, the precise spatial distribution of these pectin forms may vary among plant species (Li et al. 1994; Geitmann et al. 1995; Parre and Geitmann 2005; Hou et al. 2021). According to our findings in the control group, methyl-esterified pectins in *C. sinensis* were localized at the pollen tube tip, whereas de-esterified acidic pectins were distributed along the entire pollen tube. Taken together, these results indicate that the distribution patterns of methyl-esterified and de-esterified acidic pectins in the pollen tube wall exhibit species-specific differences, which may be associated with species-dependent regulation of the degree of pectin methyl-esterification. A partial overlap between JIM5 and JIM7 signals at the pollen tube tip was also observed under control conditions in our study. Such overlap may reflect the dynamic balance between secretion of highly methyl-esterified pectins and their rapid modification by pectin methylesterases during active pollen tube growth, particularly under in vitro conditions. It should be noted that PI staining labels negatively charged polysaccharides in the newly deposited cell wall matrix and is therefore not strictly specific to pectins. Consequently, PI and JIM7 signals are not expected to completely overlap.

Alterations in the distribution of methyl-esterified and de-esterified acidic pectins within the pollen tube wall under environmental stress conditions have been previously reported. In *C. sinensis*, Wang et al. (2016) demonstrated that methyl-esterified pectins were localized exclusively at the apical region under control conditions, but disappeared from the apex and became localized near the germination aperture following low temperature stress. In contrast, in the present study, the accumulation of methyl-esterified pectins at the pollen tube tip showed a significant increase relative to the control only after the 15 °C treatment. It is well established that the accumulation of methyl-esterified pectins at the pollen tube tip supports pollen tube elongation by maintaining cell wall elasticity (Bosch and Hepler 2005; Chebli and Geitmann 2007). Accordingly, the increase observed at 15 °C may indicate the presence of an adaptive response aimed at enhancing apical cell wall elasticity to sustain pollen tube elongation under low temperature conditions.

On the other hand, the more pronounced alterations in pectin distribution observed in both the apical and neck regions following the 5 °C treatment, compared with the other groups, suggest that pectins may have adopted a more rigid configuration as a result of increased pectin methylesterase activity. This interpretation is also consistent with the markedly reduced pollen tube lengths observed in this group. Similarly, Bilska-Kos et al. (2017) reported that low temperature stress induced substantial changes in pectin levels and in the activities of enzymes associated with pectin metabolism in *Zea mays* pollen tubes. Wang et al. (2016) demonstrated that de-esterified acidic pectins were absent from the apical region of pollen tubes under control conditions in *C. sinensis*, but gradually increased toward the neck region. However, the same study reported that following low temperature stress, de-esterified acidic pectins were distributed throughout the entire pollen tube, including the apical region. In agreement with these findings, our study revealed that significant changes in de-esterified acidic pectin distribution at both the apical and neck regions were observed only after the 5 °C treatment. Taken together, these results suggest that severe low temperature stress may suppress pollen tube growth by promoting cell wall stiffening.

CFW stains β-glucans in general and is therefore not strictly specific to cellulose, as it can also label callose. Consequently, the fluorescence signal detected with CFW likely reflects the combined contribution of cellulose and callose within the pollen tube wall. In untreated pollen tubes of *Nicotiana tabacum* and *C. sinensis*, cellulose has been reported to be distributed along the entire pollen tube wall, including the apical region, and no significant changes in cellulose distribution at the apex and neck regions were observed following low temperature stress (Parrotta et al. 2019; Çetinbaş-Genç et al. 2020). It is well established that cellulose synthase (CESA) complexes are predominantly localized at the apical region, with lower abundance in the neck region (Cai et al. 2011). In light of these findings, the increased accumulation of cellulose at the apex observed in our study, despite the largely unchanged cellulose profile along the pollen tube, suggests that low temperature stress may enhance CESA activity without markedly altering its localization. This interpretation is consistent with reports indicating that environmental stress conditions may only partially affect the membrane localization of CESA complexes (Cai et al. 2011; Parrotta et al. 2019). Considering that low temperature reduces membrane fluidity, thereby negatively affecting vesicle trafficking and cell wall elasticity (Quinn 1988; Bosch and Hepler 2005; Parrotta et al. 2019), the increased cellulose accumulation at the apical region may represent a stabilizing response that supports apical integrity under stress conditions. Moreover, the relatively slower response of CESA complexes to environmental stresses compared with pectin metabolism (Kovacs and Keresztes 2002) may explain why cellulose distribution along the pollen tube exhibited more limited changes than pectin distribution across all treatment groups.

Various stress factors have been reported to induce callose accumulation at the pollen tube apex, which can reduce pollen tube elongation and reproductive success (Wang et al. 2003). However, the spatial pattern of callose deposition under low temperature stress may vary among species (Çetinbaş-Genç et al. 2019; Parrotta et al. 2019). In the present study, the increase in callose accumulation at the apical region observed after all treatments is consistent with previous findings indicating that callose deposition at the apex reflects a slowdown or arrest of pollen tube elongation, and it provides an explanation for the observed reductions in pollen tube length (Hao et al. 2013). Notably, the more pronounced alterations in callose distribution at both the apical and neck regions observed after the 5 °C treatment suggest that this temperature induces increased rigidity of the pollen tube wall. When considered together with the marked changes observed in the distribution of methyl-esterified and de-esterified acidic pectins in the same group, these findings indicate that enhanced cell wall stiffening at 5 °C plays a key role in limiting pollen tube elongation.

ROS play a central role in pollen tube growth regulation as well as in oxidative stress responses, and under stress conditions, elevated ROS levels are balanced by enzymatic antioxidants such as SOD, CAT, and APX, together with non-enzymatic antioxidants including phenolics and flavonoids (Foreman et al. 2003; Smirnova et al. 2014). High SOD activity has been reported to support pollen viability and germination by maintaining oxidative balance (Ren et al. 2021). However, in the present study, a significant increase in SOD activity relative to the control was observed only after the 10 °C treatment, despite concomitant reductions in pollen germination rate and pollen tube length. In contrast, the absence of a change in SOD activity following the 5 °C treatment suggests that the severity of stress at this temperature may have metabolically suppressed antioxidant defense responses. Similar findings have been reported in *Corylus avellana* (Çetinbaş-Genç et al. 2019) and in *Mangifera indica*, where cultivar-dependent variability in SOD responses was observed (Liu et al. 2023). Taken together, these results indicate that SOD responses under low temperature stress may vary depending on stress intensity and duration, as well as on the cellular metabolic capacity.

It has been reported that plants tolerant to freezing, cold, or heat stress exhibit increased activities of ROS-scavenging enzymes such as CAT and APX (Saruyama and Tanida 1995; Suzuki and Mittler 2006). In the present study, CAT activity remained unchanged across all treatment groups, whereas APX activity increased significantly following the 10 °C and 5 °C treatments. This finding suggests that, in *C. sinensis* pollen, elevated levels of H₂O₂ under low temperature conditions are primarily detoxified via the ascorbate-glutathione cycle through APX, while CAT may become operative only at higher H₂O₂ thresholds. This interpretation is consistent with the enzymatic properties of CAT and APX, whereby CAT exhibits low affinity but high capacity and is therefore effective at elevated H₂O₂ concentrations, whereas APX displays high efficiency even at low H₂O₂ levels (Mhamdi et al. 2010; Foyer and Noctor 2011). Similarly, it has been reported that a 4 °C treatment did not alter CAT activity in *Corylus avellana* (Çetinbaş-Genç et al. 2019), whereas low temperature stress increased CAT activity in *Keteleeria fortunei* (Liu et al. 2020). Together, these findings indicate that CAT and APX responses to low temperature stress may vary depending on stress intensity, duration, and tissue-specific metabolic capacity (Köhler et al. 2017).

In the present study, the reduction in DPPH radical scavenging capacity observed only after the 5 °C treatment suggests that this temperature induces an oxidative stress level that exceeds the antioxidant defense capacity of pollen grains. Similarly, it has been reported that when environmental stress surpasses a critical threshold, non-enzymatic antioxidant reserves are rapidly depleted, resulting in pronounced decreases in DPPH activity (Pressman et al. 2006; Djanaguiraman et al. 2014). Although an increase in phenolic and flavonoid accumulation following low temperature exposure, particularly at 5 °C, has been reported in *Citrus species* (Mohammadrezakhani et al. 2018), our results demonstrated a decrease in these compounds across all temperature treatments, with the lowest levels detected at 5 °C. These discrepancies indicate that phenolic and flavonoid responses to low temperature stress can vary substantially depending on species, cultivar, and physiological status (Sanghera et al. 2011; Lukatkin et al. 2012).

The ability of plants to cope with stress has been suggested to be closely associated with HSPs (Simoes-Araujo et al. 2003; Brown et al. 2016). In the present study, the higher levels of HSP70 detected in the cytosolic fraction compared with the membrane fraction suggest that this protein predominantly performs its primary functions in the cytosol. This observation is consistent with reports indicating that protein folding disturbances under low temperature stress primarily arise in the cytosolic compartment (Haslbeck and Vierling 2015). Increased HSP70 levels have also been reported in pollen grains of *Paeonia lactiflora* and *Magnolia denudata* following cryopreservation treatments (Ren et al. 2019). In our study, the increase in HSP70 levels in the membrane fraction observed exclusively after the 5 °C treatment suggests that this temperature imposes a more severe stress on membrane integrity and fluidity, thereby promoting the recruitment of HSP70 to the membrane to exert its protective function. This finding is consistent with previous studies indicating that, depending on the severity of low temperature stress, HSP70 may undergo redistribution among intracellular fractions rather than exhibiting a uniform induction response (Kollipara et al. 2002; Lee et al. 2009; Dumont et al. 2011; Hlaváčková et al. 2013).

In pollen tubes, both HSP70 and SuSy can occur in cytosolic and membrane-associated forms. The cytosolic form of SuSy mainly supports general carbohydrate metabolism, whereas the membrane-associated form supplies UDP-glucose required for cellulose and callose synthesis (Wang et al. 2014; Parrotta et al. 2016). Similarly, cytosolic HSP70 is primarily involved in protein folding and proteostasis, while membrane-associated HSP70 contributes to the stabilization of cellular membranes under stress conditions (Lee et al. 2009; Hlaváčková et al. 2013). Therefore, differences observed between these fractions may reflect functional redistribution of these proteins in response to stress. An association between SuSy levels in pollen grains and pollen sensitivity to stress conditions has been previously reported (Pressman et al. 2006). In maize, rice, and tobacco, both cytosolic and membrane-associated SuSy activities have been shown to change markedly under drought and heat stress, and these alterations have been linked to pollen stress sensitivity (Mu et al. 2009; Parrotta et al. 2016; Li et al. 2022). In the present study, the decrease in membrane-associated SuSy levels at 15 °C and 10 °C, together with the increase observed at 5 °C, suggests that UDP-glucose required for cellulose and callose synthesis is directly redirected toward the cell wall. This interpretation is consistent with the high callose and cellulose fluorescence signals detected in the 5 °C group. In contrast, the overall reduction in cytosolic SuSy levels indicates that under low temperature stress, sucrose metabolism is shifted away from growth-oriented processes supporting pollen tube elongation toward mechanisms associated with survival and stress tolerance. This interpretation is in agreement with previous studies reporting that, under abiotic stress conditions, carbon metabolism is reprogrammed to prioritize the maintenance of cellular integrity (Ruan 2014; Thalmann and Santelia 2017).

It has been reported that, in multivariate analyses, evaluating stress severity based on the cumulative magnitude of changes rather than on individual parameters yields more reliable outcomes (Hasanuzzaman et al. 2012; Scholz et al. 2020). Moreover, the clustering of stress conditions into subgroups has been suggested to reflect threshold levels at which similar defense strategies are activated (Djanaguiraman et al. 2014). Consistent with these observations, our heat map analyses revealed that the 5 °C and 10 °C treatments represented the conditions showing the greatest deviation from the control group, whereas the 15 °C group exhibited a more limited response profile.

Although pollen germination and pollen tube length decreased under all temperature treatments, the underlying cellular and biochemical responses differed depending on temperature severity. At 15 °C, reduced phenolic and flavonoid contents together with decreased cytosolic and membrane-associated HSP70 and cytosolic SuSy levels indicated metabolic weakening, while increased callose, cellulose, and methyl-esterified pectin accumulation suggested an early adaptive response reinforcing the pollen tube cell wall. At 10 °C, the induction of SOD and APX activities indicated partial activation of antioxidant defenses, whereas continued callose and cellulose accumulation pointed to structural reorganization aimed at maintaining mechanical stability. In contrast, the 5 °C treatment resulted in pronounced metabolic suppression and increased cell wall rigidification associated with enhanced callose, cellulose, and de-esterified pectin accumulation, ultimately limiting pollen tube elongation.

## Conclusion

In conclusion, low temperature stress significantly affected pollen germination and pollen tube growth in *C. sinensis*. The results demonstrate that the severity of temperature determines the balance between adaptive responses and metabolic suppression. Moderate stress conditions triggered partial antioxidant and structural responses, whereas severe low temperature promoted cell wall rigidification associated with changes in pectin dynamics and increased callose and cellulose accumulation, ultimately limiting pollen tube elongation. These findings highlight the coordinated interaction between cell wall remodeling, antioxidant systems, and stress-related proteins in shaping pollen tube responses to low temperature.

## Supplementary Information

Below is the link to the electronic supplementary material.

Supplementary file1 (DOCX 20 KB)

Supplementary file1 (JPG 20 KB)

## Data Availability

The data supporting the findings of this study were generated during the current study. All relevant data are included in the manuscript and its Supplementary Information. Additional data are available from the corresponding author upon reasonable request.
