# Supplementary material for: Low-temperature stress modulates pollen tube growth through temperature-dependent multi-level regulatory mechanisms in Camellia sinensis
Source: Plant Reprod. 2026 May 8;39(2):4. doi: 10.1007/s00497-026-00539-3 (PMC13156120; doi:10.1007/s00497-026-00539-3)
Supplement: Supplementary file 1 — Supplementary file1 (DOCX 20 KB) [file 497_2026_539_MOESM1_ESM.docx]

**SUPPLEMENTARY TABLES**

**Supplementary Table 1.** Protein concentrations of pollen extracts used for enzyme activity assays.

| **Treatment** | **Protein concentration (mg mL⁻¹)** |
| --- | --- |
| Control | 3.13 ± 0.13 |
| 15 °C | 2.57 ± 0.19 |
| 10 °C | 1.88 ± 0.23 |
| 5 °C | 1.46 ± 0.11 |

**Supplementary Table 2.** Protein concentrations of cytosolic and membrane pollen extracts used for immunoblot analyses.

| **Treatment** | **Protein concentration (mg mL⁻¹)** | |
| --- | --- | --- |
|  | Membrane | Cytosol |
| Control | 10.55 ± 1.36 | 10.42 ± 1.28 |
| 15 °C | 30.77 ± 2.02 | 10.71 ± 1.33 |
| 10 °C | 10.54 ± 1.24 | 10.48 ± 1.29 |
| 5 °C | 20.3 ± 1.76 | 10.45 ± 1.34 |

**Supplementary Table 3.** Raw dataset used for heatmap analysis.

|  | **15 ℃** | **10 ℃** | **5 ℃** |
| --- | --- | --- | --- |
| Pollen germination | 63.75 | 82.68 | 92.93 |
| Pollen tube length | 54.4189517 | 59.26 | 74.41 |
| Newly secreted cell wall material at apex | 13.64 | 17.71 | 0.77 |
| Newly secreted cell wall material along the tube | 0.38 | 0.99 | 0.47 |
| Methyl-esterified pectin at apex | 88.22 | 18.21 | 11.19 |
| Methyl-esterified pectin along the tube | 0.37 | 0.93 | 0.99 |
| De-esterified acidic pectin at apex | 5.65 | 50.73 | 73.24 |
| De-esterified acidic pectin along the tube | 0.35 | 0.27 | 0.94 |
| Callose at apex | 129.78 | 203.90 | 189.71 |
| Callose along the tube | 0.99 | 0.02 | 0.99 |
| Cellulose at apex | 130.50 | 121.42 | 120.07 |
| Cellulose along the tube | 0.06 | 0.03 | 0.19 |
| SOD activity | 47.53 | 111.03 | 63.19 |
| CAT activity | 7.10 | 53.12 | 34.40 |
| APX activity | 32.22 | 73.08 | 67.75 |
| %DPPH activity | 0.38 | 2.68 | 22.24 |
| Total phenolic compounds | 37.58 | 46.06 | 45.68 |
| Total flavonoid compounds | 20.61 | 27.19 | 40.58 |
| HSP70 membrane | 19.11 | 24.72 | 15.05 |
| HSP70 cytosol | 14.59 | 8.40 | 4.037 |
| SuSy membrane | 93.49 | 87.02 | 27.50 |
| SuSy cytosol | 82.78 | 70.41 | 47.31 |
