# Supplementary figures and images for: Low-temperature stress modulates pollen tube growth through temperature-dependent multi-level regulatory mechanisms in Camellia sinensis
Source: Plant Reprod. 2026 May 8;39(2):4. doi: 10.1007/s00497-026-00539-3 (PMC13156120; doi:10.1007/s00497-026-00539-3)

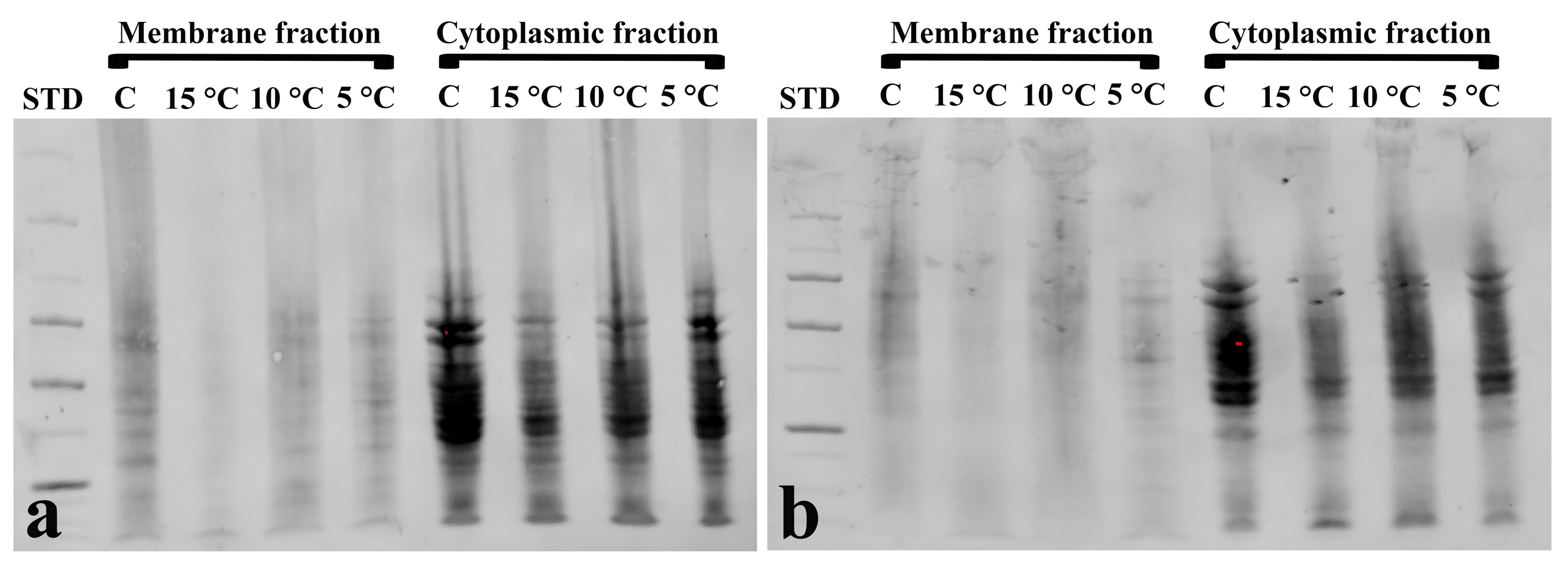

Supplement: Supplementary file 2 — Supplementary file1 (JPG 20 KB) [file 497_2026_539_MOESM2_ESM.jpg]
